# Challenges in Prenatal Ultrasound Diagnosis of Rubinstein–Taybi Syndrome: A Case Report and Comprehensive Literature Review

**DOI:** 10.3390/ijms26115142

**Published:** 2025-05-27

**Authors:** Daniela Roxana Matasariu, Iuliana-Elena Bujor, Roxana Maria Gireada, Luiza Maria Guzga, Florina Mihaela Nedelea, Monica Titianu, Alexandra Ursache

**Affiliations:** 1 Department of Mother and Child, University of Medicine and Pharmacy ‘Gr. T. Popa’, 700115 Iasi, Romania; daniela.matasariu@umfiasi.ro (D.R.M.); roxgireada@gmail.com (R.M.G.); alexandra.ursache@umfiasi.ro (A.U.); 2 Department of Obstetrics and Gynecology, Cuza Voda Hospital, 700038 Iasi, Romania; moni_titianu@yahoo.com; 3 Department of Obstetrics and Gynecology, Nativia Clinic, 030167 Bucharest, Romania; guzga.luiza@gmail.com; 4 Department of Obstetrics and Gynecology, Filantropia Clinical Hospital, 011171 Bucharest, Romania; ina.nedelea@gmail.com

**Keywords:** Rubinstein–Taybi syndrome, ultrasound, prenatal diagnosis, broad thumb-hallux, congenital anomalies, SNP array, *CREBBP* gene, chromosome 16p13.3

## Abstract

Rubinstein–Taybi syndrome (RSTS) is a rare genetic disorder characterized by distinctive craniofacial, limb, and developmental abnormalities, often identified postnatally. Prenatal diagnosis remains challenging due to a scarcity of ultrasound diagnostic markers and a wide range of phenotypic manifestations. We describe the case of a 28-year-old pregnant patient who presented to our center after fetal abnormalities such as aberrant cranial morphology, a shorter femur, and rocker-bottom feet were detected. A comprehensive ultrasound examination at 26 weeks revealed skeletal and craniofacial characteristics suggestive of RSTS, which prompted genetic counseling and molecular karyotyping. Single-nucleotide polymorphism (SNP) array analysis confirmed a loss on chromosome 16p13.3, including the *CREB-binding protein* (*CREBBP*) gene, confirming the suspicion. This case emphasizes the importance of genetic testing and sophisticated prenatal imaging in enabling an early and precise diagnosis of RSTS, offering important information on its prenatal phenotype and supporting family counseling. Extensive research becomes vital in establishing precise ultrasound markers for the early detection of RSTS during pregnancy.

## 1. Introduction

Rubinstein–Taybi syndrome (RSTS), also known as Broad Thumb-Hallux syndrome, is a rare congenital disorder characterized by a diverse range of clinical features, including intellectual disability, congenital anomalies, and an increased susceptibility to certain malignancies [1,2,3]. It is estimated to have a birth prevalence ranging from 1 in 100,000 to 125,000 live births [1,4]. While RSTS is primarily considered an autosomal dominant disorder, most cases arise from de novo mutations, with most affected individuals having healthy parents [1,2,4,5,6,7].

The clinical presentation of RSTS is variable but commonly includes postnatal growth deficiency, dysmorphic facial features (arched eyebrows, long eyelashes, down-slanting palpebral fissures, a broad nasal bridge, and a beaked nose), and microcephaly. Additional characteristic features encompass broad thumbs and halluces with radial deviation, cognitive impairment, skin abnormalities, renal anomalies, and congenital heart defects [1,2,3,4,5,8,9].

The genetic basis of RSTS involves mutations in the *CREBBP* or *EP300* genes, which encode transcriptional coactivators involved in chromatin remodeling. Both are ubiquitously expressed proteins with high sequence similarities but distinct functions. The clinical severity and phenotype can vary depending on the affected gene, with mutations in *CREBBP* being more prevalent. Mutations in these genes disrupt normal cellular pathways and gene expression, contributing to the multisystem abnormalities observed in RSTS [2,3,8,10,11,12].

Diagnosis of RSTS is primarily clinical, relying on recognizing characteristic features, although genetic testing can confirm the diagnosis in some cases. There are currently no specific diagnostic criteria available [1,3,8,10,11]. Prenatal diagnosis is rare and challenging, with few cases identified prenatally based on ultrasound findings of limb anomalies [4,13,14,15].

Management of RSTS involves multidisciplinary care to address the various medical and developmental needs of affected individuals [2,11]. Additionally, due to the increased risk of malignancies, lifelong surveillance for tumor development is recommended [2,11,16,17].

Due to the challenges inherent in prenatal diagnostic procedures for RSTS, we believe that the case presented in the subsequent section will contribute to the formulation of specific diagnostic criteria.

## 2. Materials and Methods

### 2.1. Case Presentation

A 28-year-old pregnant woman presented at our center for further evaluation following the identification, during prenatal investigations, of fetal anomalies, including an abnormal cranial shape, a shortened femur, and rocker-bottom feet.

The patient had a history of pregnancy loss at 8 weeks of gestation and was diagnosed with hereditary thrombophilia, being a carrier of three genetic heterozygous mutations (Factor V Leiden, Factor II, and methylenetetrahydropholate reductase (MTHFR) C677T). The patient’s family history revealed no reported cases of thrombotic events, thrombophilia, consanguinity, congenital malformations, or chromosomal anomalies.

**Figure 1:**
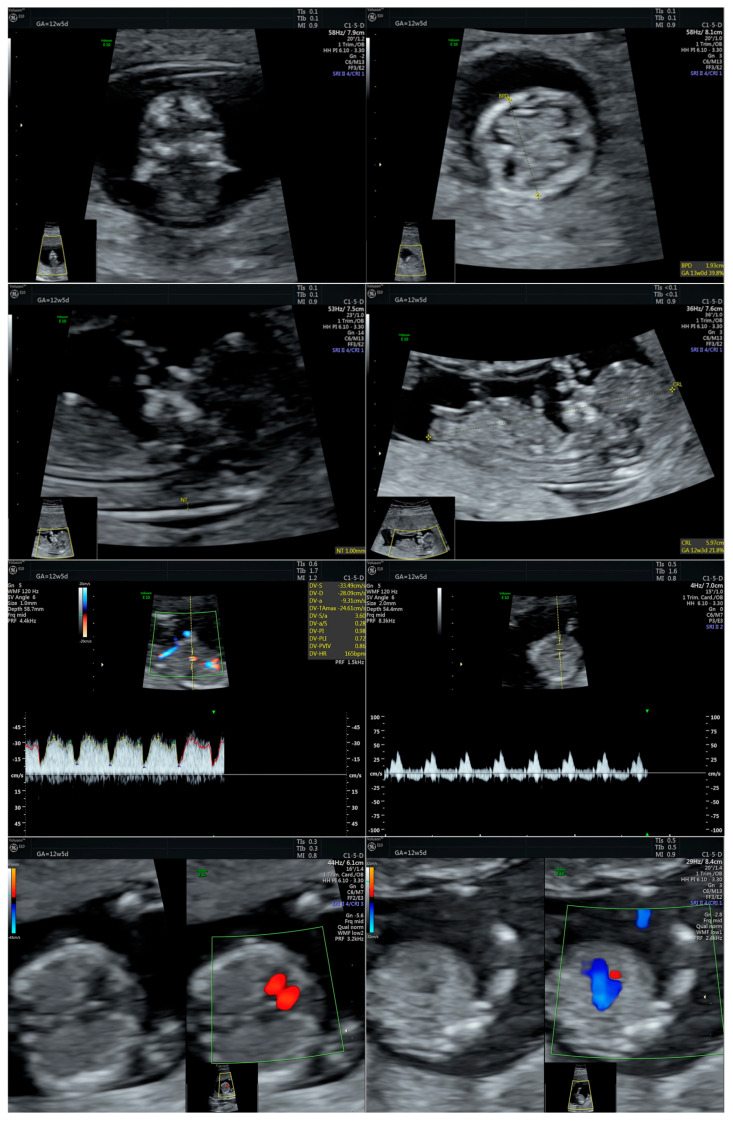
Series of first-trimester morphological ultrasound images of the fetus at 12 weeks of gestation, with normal appearance (the first two images display sagittal and coronal sections of the fetal head; the third image shows a sagittal view, highlighting the presence of the nasal bone and the thickness of the nuchal translucency; the fifth image shows the Doppler assessment of the ductus venosus with normal flow; the sixth image displays the Doppler evaluation of the tricuspid blood flow showing no regurgitation (normal appearance); the seventh and eighth images illustrate fetal cardiac assessment with four-chamber view, as well as the aorta and pulmonary artery connected by the ductus arteriosus). Abbreviations: BPD (Biparietal diameter); NT/NF (Nuchal translucency/Nuchal fold); GA (Gestational age); CRL (Crown-rump length); HR (Heart rate); PI (Pulsatility index); w (weeks); d (days).

The current pregnancy was spontaneously conceived. Following the diagnosis of thrombophilia, the hematologist advised the patient to undergo daily low-molecular-weight heparin treatment throughout the pregnancy. At 12 weeks and 5 days, the patient underwent a first-trimester screening morphological ultrasound, revealing a fetus with normal development, consistent with gestational age, with no identified abnormalities (Figure 1). The combined first-trimester screening test showed a low risk for aneuploidy, with a final risk of 1:14,000 for Down Syndrome and 1:100,000 for trisomies 13 and 18. The multiple of the median (MoM) values were 4.13 for Β human chorionic gonadotropin (hCG) and 2.86 for pregnancy-associated plasma protein A (PAPP-A).

**Figure 2:**
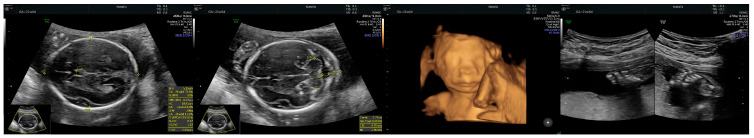
Series of second-trimester morphological ultrasound images of the fetus at 21 weeks of gestation, with normal appearance (the first image shows a transthalamic view of the fetal head, while the second one shows a transcerebral view; the third one is a 3D assessment of the fetal face, and the final image shows the sole of the fetal foot). Abbreviations: BPD (Biparietal diameter); HC (Head circumference); CM (cisterna magna); Cereb (Cerebellum); NF/NT (nuchal fold); GA (Gestational age); CSP (cavum septum pellucidum); EFW (Estimated fetal weight); w (weeks); d (days).

Subsequent ultrasound evaluations revealed a fetus with normal development, and the second-trimester ultrasound for fetal anomaly screening, performed at 21 weeks and six days, did not identify any pathological findings (Figure 2) (Table 1).

At 26 weeks and 2 days, a routine ultrasound examination revealed several alarming characteristics, including a femur shorter than the third percentile, rocker-bottom feet, and a triangular-shaped skull. In light of these results, prenatal diagnosis was advised and accepted by the family to identify a potential genetic cause of the above-mentioned anomalies (Table 1).

### 2.2. Case Management

**Figure 3:**
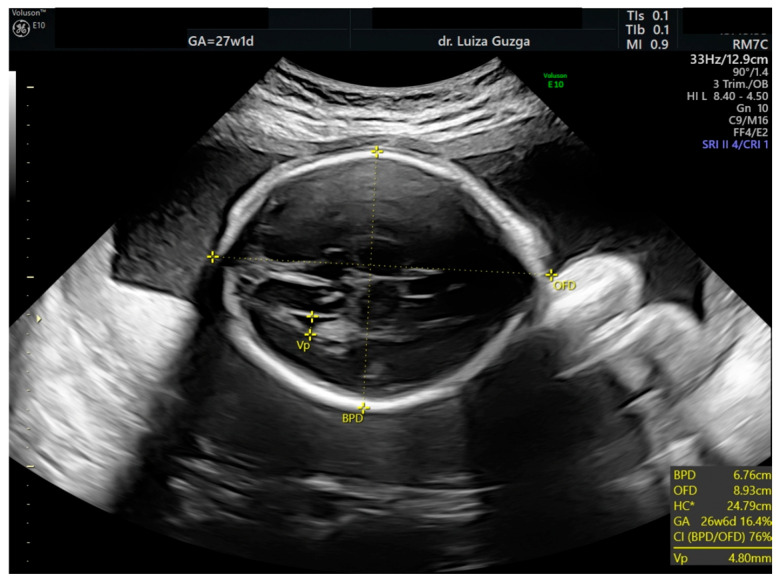
Two-dimensional ultrasound examination of the fetus at 27 weeks of gestation, revealing the abnormal shape of the head (trigonocephaly). Abbreviations: BPD (Biparietal diameter); HC (Head circumference); GA (Gestational age); Vp (Posterior ventricle); w (weeks); d (days).

**Figure 4:**
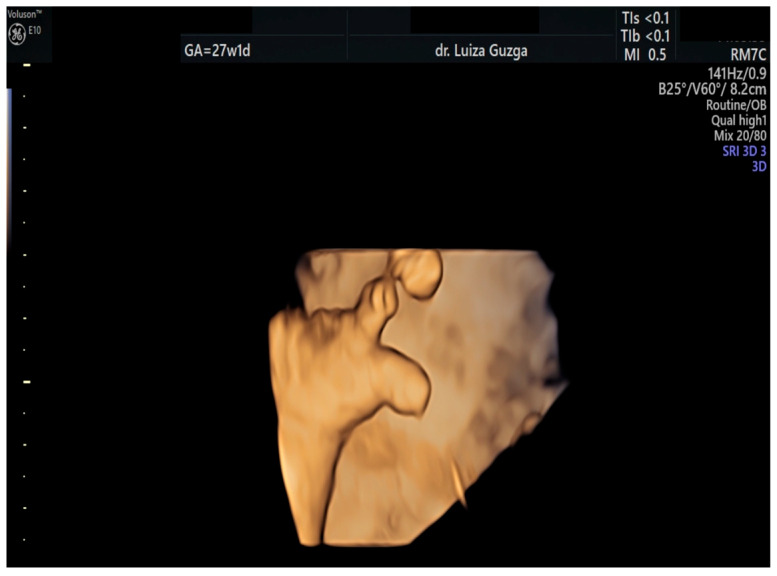
Three-dimensional ultrasound assessment of the fetal hand at 27 weeks of gestation, revealing a short thumb. Abbreviations: GA (gestational age); w (weeks); d (days).

**Figure 5:**
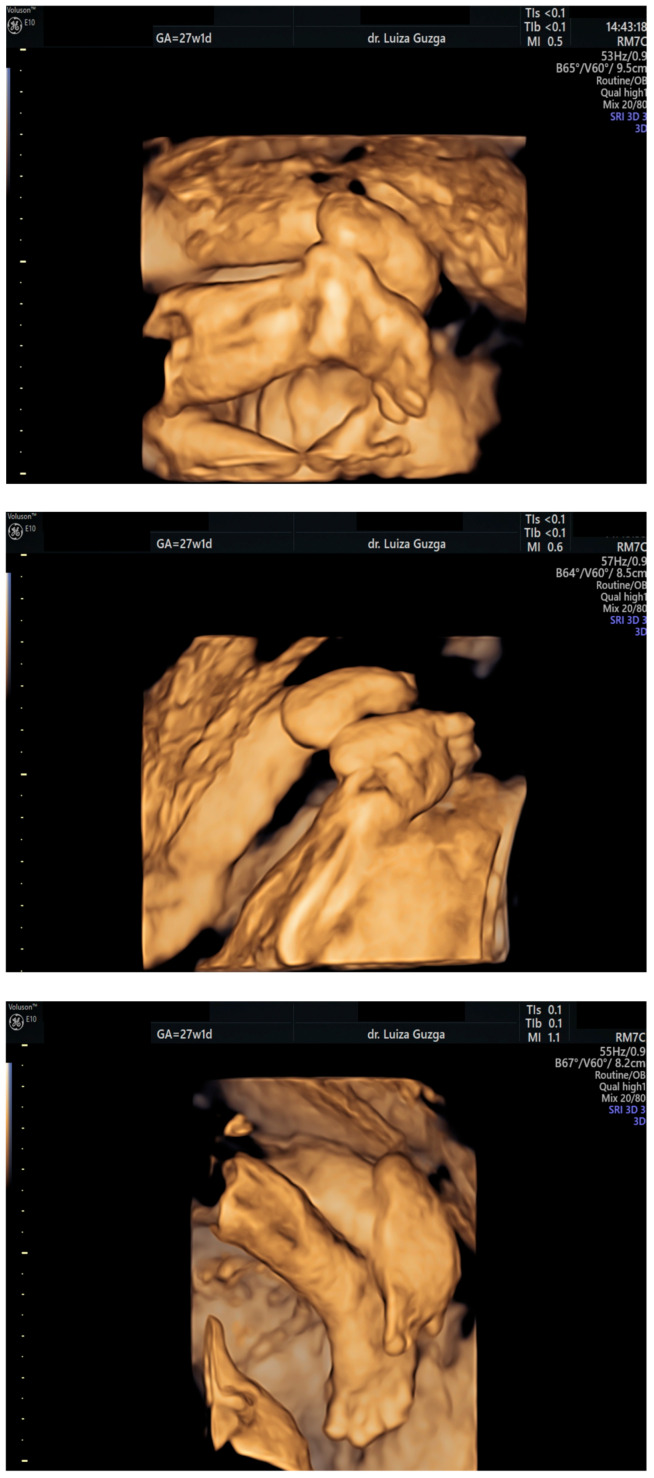

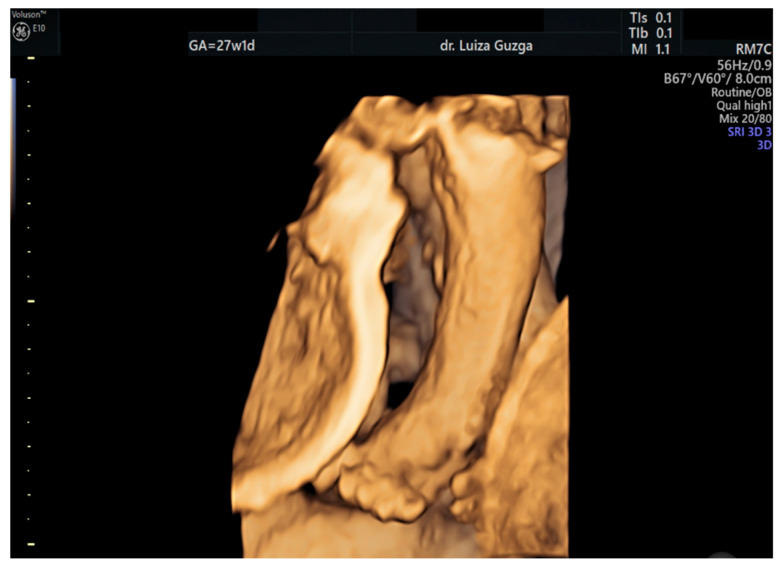
A series of 3D ultrasound assessments of the fetal lower limbs at 27 weeks of gestation, indicating rocker-bottom feet. Abbreviations: GA (gestational age); w (weeks); d (days).

**Figure 6:**
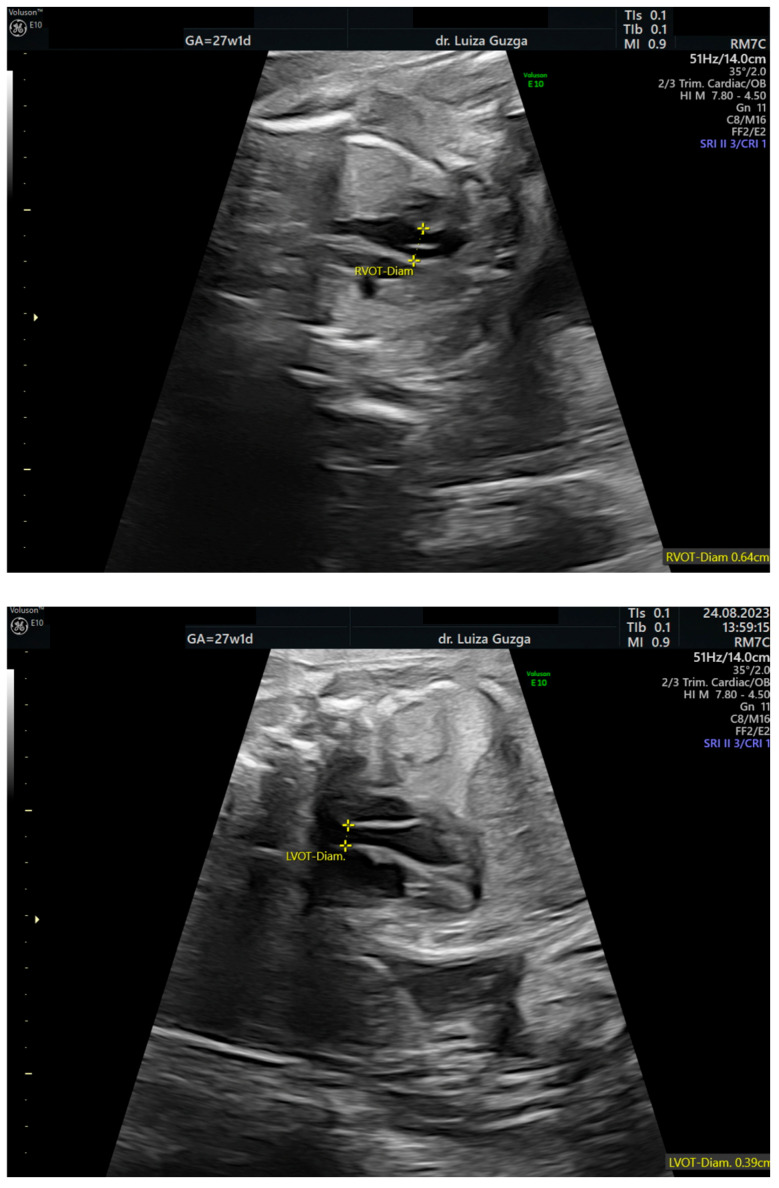
Two-dimensional fetal cardiac assessment of the outflow tracts (LVOT—left ventricular outflow tract; RVOT—right ventricular outflow tract) at 27 weeks of gestation, revealing a hyperechoic pulmonary valve. Abbreviations: GA (gestational age); w (weeks); d (days).

**Figure 7:**
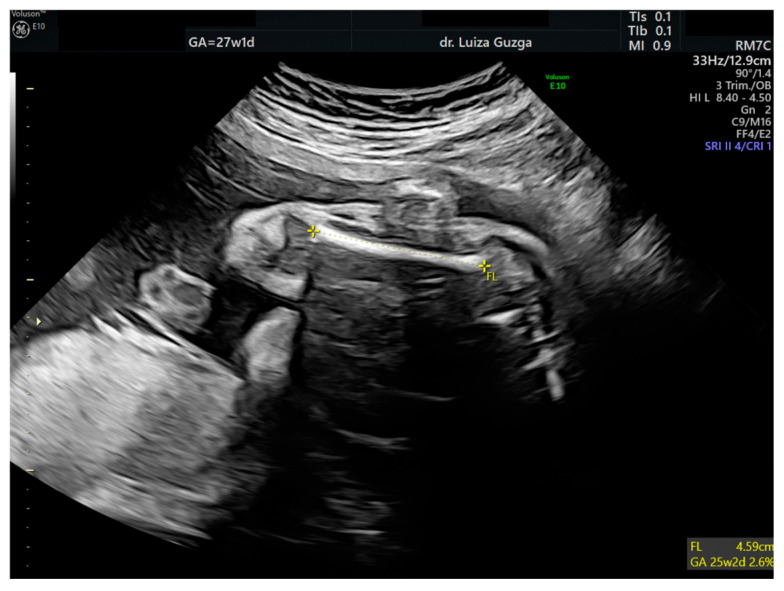
Two-dimensional ultrasound assessment of the fetus at 27 weeks of gestation, identifying a short femur. Abbreviations: FL (femur length); GA (gestational age); w (weeks); d (days).

At 27 weeks and one day, the patient was evaluated by a maternal–fetal medicine specialist. We performed serial examinations using 2D and 3D ultrasounds with a General Electric Voluson^TM^ E10 (GE Health Care, Chicago, IL, USA) machine equipped with an abdominal RM7C 2–6 MHz and RM6C convex probe and a vaginal RIC MHz probe. The ultrasound examination confirmed the presence of an abnormal head shape (trigonocephaly), short thumb, rocker-bottom feet, a hyperechoic pulmonary valve with normal function, and a short femur (Figure 3, Figure 4, Figure 5, Figure 6 and Figure 7 and Table 1).

The patient was advised to undergo amniocentesis and genetic testing. Quantitative fluorescent polymerase chain reaction (QF-PCR) genetic testing did not identify aneuploidies for chromosomes 13, 18, 21, X, or Y, and also determined the genetic sex of the fetus to be male.

**Figure 8:**
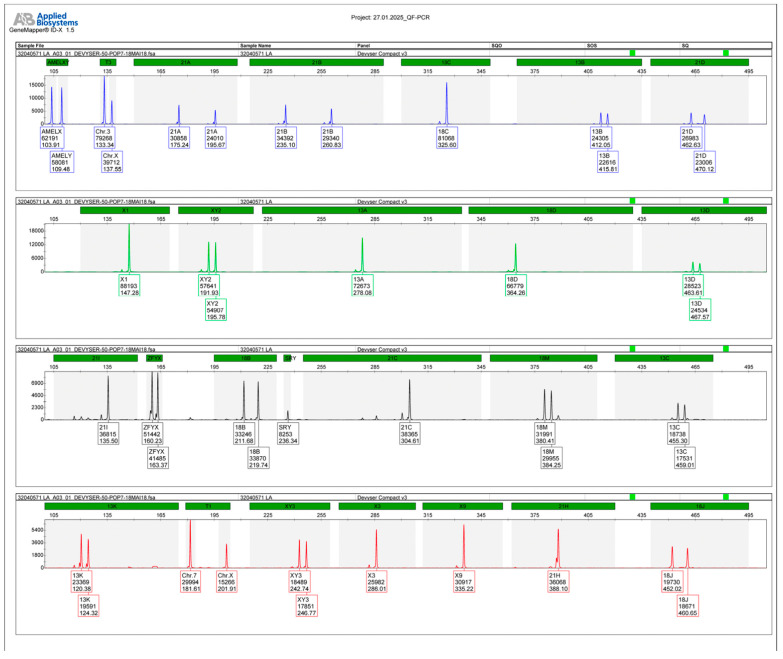
Molecular karyotype (SNP array) analysis shows a copy number loss at chromosomal location 16p13.3, including the *CREBBP* gene, consistent with an RSTS diagnosis.

Molecular karyotyping using a single-nucleotide polymorphism array (SNP array) revealed a copy number loss at chromosomal location 16p13.3, described as arr[GRCh38] 16p13.3(3738866_4570283)x1, highlighting a male karyotype and the molecular genetic analysis (as indicated by the AMELX/AMELY peaks and the presence of a single X and Y chromosome) with a heterozygous deletion of approximately 831 kb on the short arm of chromosome 16, encompassing 11 *OMIM* genes, including the *CREBBP* gene (haploinsufficiency score 3), which is responsible for RSTS. The deleted segment plays a pivotal role in transcriptional regulation through histone acetylation and is crucial for normal cognitive and developmental processes. The haploinsufficiency score of 3 in the *CREBBP* gene signifies a high likelihood that a single functional copy of the gene is insufficient to maintain normal function, providing a clear molecular explanation for the clinical features consistent with RSTS. Furthermore, the peak patterns for chromosomes 13,18, and 21 show normal disomic profiles (balanced biallelic ratios), which effectively rules out trisomies. These findings support the observation that the clinical phenotype corresponds to the submicroscopic deletion at 16p13.3, confirmed with the SNP array. This genomic alteration and its potential clinical implications are further illustrated in Figure 8, which visualizes the chromosomal region affected and highlights the genes encompassed by the deletion.

Following the results, the patient sought treatment at a specialized facility overseas, received genetic counseling, and made the decision to end the pregnancy.

## 3. Literature Search: Methodology and Results

We reviewed the literature by searching PubMed, Embase, and Medline databases for studies on the prevalence of RSTS cases. We selected the most relevant articles/studies, including case reports, systematic reviews, and meta-analyses. We identified 14 instances [3,4,13,14,17,18,19] with prenatal ultrasound signs and diagnostic suspicions of RSTS; of these, only three cases were diagnosed with RSTS during pregnancy, and one case reported no data on the timing of the diagnosis (prenatal or postnatal) (Table 2).

## 4. Discussion

RSTS is associated with diagnostic challenges due to its variable presentation, often leading to a postnatal only establishment of the diagnosis. Prenatal suspicion is rare.

### 4.1. Clinical Manifestations

Clinical features, such as broad thumbs and distinctive facial characteristics, may suggest RSTS. Establishing the diagnosis typically relies on molecular analysis, which confirms about 56% of cases [15,18]. Rarely, prenatal detection occurs through advanced imaging techniques, although molecular analysis is usually deferred [1,15]. RSTS presents a challenging prognosis marked by intellectual disability, growth retardation, and an elevated tumor risk [1,11,18]. Cranial malformations, including microcephaly and delayed anterior fontanel closure, are common. In contrast, posterior fossa anomalies like Dandy–Walker malformation (DWM) are less frequent, but can occur [1,11,15]. RSTS manifests with a wide spectrum of clinical features affecting multiple organ systems, including skeletal anomalies, neurological manifestations, and behavioral disorders, but has characteristic facial and limb anomalies [1,3,11,14,19,20,21]. Novel presentations, such as midline notches of the upper lip, median grooves of the lower lip, and brachydactyly, contribute to completing the wide range of features that RSTS exhibits [21].

### 4.2. Genetics of the Rubinstein–Taybi Syndrome (RSTS)

Reports in the literature indicate that the variant responsible for the disease is detected in 37–75% of individuals with the classical form of the syndrome, and in about 25% of those with an incomplete phenotype [3,22]. Two models explain how mutations in the *CREBBP* gene may lead to the manifestations of RSTS haploinsufficiency and dominant negative effects. In haploinsufficiency, one functional copy of *CREBBP* cannot fulfill all the cell’s normal development and function requirements. At the same time, in the dominant negative effect, the abnormal protein produced by mutant alleles inhibits the standard functional protein of the healthy allele. Incomplete RSTS exhibits a wide range of phenotypic manifestations, with two extreme expressions noted [13,23]. One extreme is incomplete RSTS, initially described by Cotsirilos et al. in 1987, where affected individuals show features similar to RSTS but with normal intelligence [23]. Cases previously labeled as Rubinstein-like syndrome have been reclassified as incomplete RSTS. On the other end of the spectrum is severe RSTS, described by Bartsch et al. in patients with extensive deletions in chromosome 16p13.3 [13]. These patients exhibit severe developmental abnormalities, with some experiencing early mortality. A de novo paracentric inversion of chromosome 16-inv(16) (p13.3.;q13) has also been reported in some patients with RSTS, affecting the *CREBBP* gene area, and can be confirmed in a significant percentage of cases using combined cytogenetic and molecular techniques [2,8,11,13,16,23,24]. There are two types of RSTS: a milder phenotype associated with *EP300* mutations, and a second type associated with *CREBBP* mutations [3,25,26]. Despite the second one being more severe, microcephaly is more common in *EP300*-mutated patients [3,25]. No significant phenotype–genotype correlation exists between *CREBBP* or *EP300* mutations in RSTS patients [3]. RSTS is linked to the interstitial 16p13.3 deletion involving the *CREBBP* gene in around 10% of cases, increasing the risk of pediatric cancers such as medulloblastomas (MBs) [27]. *CREBBP* plays a significant role in oncogenesis, with inactivation associated with increased tumor penetrance and dissemination. Somatic mutations in *CREBBP* and germline deletions are implicated in the occurrence of MBs. Histone modification, regulated by *CREBBP*, is crucial in MB pathogenesis, warranting further investigation into *CREBBP*’s role in aggressive subtypes [27].

The rarity and variability of RSTS presentations make prenatal diagnosis extremely difficult, emphasizing the importance of considering RSTS in cases with characteristic features for appropriate genetic counseling [3,11,15,21]. Although recurrence risk is generally low, sporadic cases predominate [11].

### 4.3. Prenatal Diagnosis and Differential Diagnosis of Rubinstein–Taybi Syndrome (RSTS)

- Diagnostic Challenges

Prenatal diagnosis of RSTS is rare, with only a few reported cases in the literature [3,4,14,20]. Prenatal signs of RSTS are not straightforward, complicating ultrasound diagnosis [4,14,28]. Ultrasonographic scans may not always detect hand and foot malformations associated with RSTS, making diagnosis challenging [4]. Combining 2D and 3D imaging may improve visualization of extremities, aiding in diagnosis. Specific sonographic markers, such as broad, abducted thumbs and toes, as well as distinctive facial features, may raise suspicion of RSTS [4]. Unique case presentations can aid in expanding the understanding of RSTS diagnosis. Facial and limb abnormalities, including a beaked nose, micrognathia, and broad, severely abducted thumbs and toes, can prompt suspicion of RSTS [4,14,28]. These features may be relatively specific sonographic markers for the syndrome, particularly when observed alongside polyhydramnios [14].

Genetic research on RSTS has primarily focused on mutations in the genes encoding *CREBBP* and *EP300*. Both encode homologous transcriptional coactivators that share conserved domains, including the enzymatic histone acetyltransferase (HAT) domain, which plays a critical role in chromatin remodeling. Reported genetic alterations include point mutations (nonsense, missense, frameshift, and splice-site), large deletions, translocations, and inversions. Given this genetic heterogeneity, comprehensive molecular diagnosis of RSTS becomes a challenge for practitioners. Whole-exome sequencing (WES), which uses a shotgun-based next-generation sequencing approach, is effective in identifying single-nucleotide variants and small indels, particularly within coding regions. However, its ability to detect structural variants and large deletions is limited due to short-read sequencing constraints. Multiplex ligation-dependent probe amplification (MLPA), in contrast, is well suited for detecting copy number variations (CNVs) and larger genomic deletions, though it cannot identify small sequence-level variants. Perhaps a combination of the WES-MLPA approach might overcome the limitations of each of these individual methods and enhance diagnostics. The literature underlines the value of using complementary genomic tools in diagnosing genetically heterogeneous syndromes like RSTS [28]. While there is limited research specifically on circulating micro-ribonucleic acids (miRNAs) in RSTS, miRNAs play crucial roles in gene regulation and have been implicated in various neurodevelopmental disorders. For instance, in neurodegenerative diseases, miRNAs such as miR-9, miR-181c, and miR-212 have been shown to modulate histone acetylation and phosphorylation, processes also relevant to RSTS pathology. The mutations responsible for this syndrome lead to altered transcriptional regulation, which could affect miRNA expression profiles; therefore, investigating circulating miRNAs in RSTS patients might reveal potential biomarkers for diagnosis or disease progression [29]. Proteomic analyses in RSTS are scarce; however, given the role of *CREBBP* and *EP300* in histone acetylation and transcriptional regulation, it is plausible that their dysfunction could lead to altered protein expression profiles. Proteomic studies could identify differentially expressed proteins in RSTS patients, providing insights into disease mechanisms and potential biomarkers. However, challenges such as the complexity of the plasma proteome and the need for advanced mass spectrometry techniques must be addressed to enhance the sensitivity and specificity of proteomic analysis [30]. Further research focusing on these molecular aspects may provide valuable diagnostic tools and therapeutic targets for RSTS.

- Differential diagnosis

Differential diagnoses include craniosynostosis syndromes and other genetic syndromes, such as Cornelia De Lange or Floating Harbor syndrome [4,31]. However, distinguishing RSTS from overlapping syndromes remains challenging [14,21,31].

### 4.4. Complications

Complications in RSTS patients vary and may include immune deficiencies that contribute to frequent infections, skeletal abnormalities such as short stature and dorsal kyphosis, ocular abnormalities such as congenital glaucoma, and hematological disorders [25]. While some complications, such as neural tumors and spinal cord malformations, may not be present in all cases, hematological monitoring is advised due to the increased risk of malignancies in RSTS patients [25].

### 4.5. Management

Surveillance protocols encompass various systems and concerns, including growth monitoring, neurological assessments, and tumor surveillance [2]. Regular follow-up is crucial to address emerging medical issues and support developmental progress [18]. Management strategies are primarily symptomatic, aiming to improve quality of life and address individual symptoms [11]. Where growth differs from expected [2], special attention is needed to detect growth hormone deficiency. Neurological assessments, developmental progress, and educational needs should be monitored closely alongside neurobehavioral and psychiatric assessments for conditions such as anxiety, attention deficit hyperactivity disorder (ADHD), autistic spectrum disorders (ASDs), aggression, and self-injury [2,5,8]. RSTS exhibits increased tumor susceptibility. The exact cause of this predisposition remains unknown, but it is hypothesized that the lack of tumor suppressor activity of *CREBBP* may contribute to the development of malignancies [5,17].

### 4.6. Ethical Considerations

The termination of a pregnancy following genetic confirmation of a condition such as RSTS is a deeply sensitive and ethically complex decision. Ethical considerations in such cases typically revolve around the autonomy of the parents, the potential quality of life of the child, and the role of genetic counseling in guiding these decisions. Parents have the right to make informed decisions regarding the future of their pregnancy, particularly when faced with a diagnosis of a serious genetic disorder. Ethical principles emphasize respecting the autonomy of parents to make choices that align with their values, beliefs, and understanding of their circumstances, taking into account the potential challenges they may face due to severe intellectual disabilities, physical abnormalities, and lifelong care. The role of genetic counseling is crucial in such scenarios. Genetic counselors provide families with accurate information about the condition, its genetic basis, inheritance patterns, and potential outcomes. They address the emotional aspects of the decision-making process, offer psychological support, and discuss the implications of various choices [32].

RSTS presents significant diagnostic challenges due to its broad phenotypic variability and limited prenatal diagnostic features. Recent advances in imaging techniques, along with an improved understanding of genetic and phenotypic heterogeneity, have facilitated better recognition of the syndrome. Nevertheless, distinguishing RSTS from clinically overlapping syndromes and identifying atypical or novel presentations remain important areas of ongoing research and clinical interest. Further studies are needed to clarify genotype–phenotype correlations, improve diagnostic precision, and enhance prognostic assessments.

Our case contributes to the refinement of prenatal diagnostic criteria by identifying specific ultrasound findings (such as a short femur, rocker-bottom feet, a hyperechoic pulmonary valve, and subtle craniofacial anomalies such as trigonocephaly) that may be suggestive of RSTS even before genetic confirmation. These findings, when viewed in combination, may serve as early sonographic indicators warranting targeted genetic testing in suspected cases. Moreover, this case emphasizes the value of integrating detailed fetal imaging with molecular diagnosis to support earlier and more accurate recognition of rare syndromes such as RSTS.

In terms of future directions, our case underscores the need for systematic aggregation and analysis of prenatal imaging features in genetically confirmed RSTS cases. Such efforts could lead to the development of standardized sonographic criteria, improve differential diagnosis from phenotypically similar syndromes, and potentially guide clinical decision-making during pregnancy. We believe that reporting and analyzing rare but well-documented cases such as this one will be essential in shaping more robust prenatal diagnostic frameworks for RSTS.

## 5. Conclusions

RSTS poses diagnostic challenges due to its variable presentation and scarce prenatal diagnostic features. Distinguishing RSTS from overlapping syndromes and identifying novel presentations remain areas of ongoing research and clinical interest. Early recognition, comprehensive evaluation, and multidisciplinary management are crucial for optimizing outcomes in RSTS patients. We believe that our case presentation will help establish ultrasound diagnostic suspicion criteria for this extremely rare genetic syndrome with broad phenotypic manifestations.

## Figures and Tables

**Table 1:** Prenatal ultrasound signs in our case.

Prenatal Ultrasound Findings	21 + 6	26 + 6	28 + 6	Frequency of Postnatal Findings in the Literature
Pointed forehead/premature closure of the metopic suture	-	+	+++	?
Hypotelorism	-	+	+++	Rather hypertelorism
Micrognathia/small chin size	-	+	+++	+++
Low-set ears	-	-	+	+++
Corpus callosum hypoplasia	- 19.9 mm > 5% (19.54 mm)	+ 27.7 mm < 5% (29.91 mm = 5%)	Difficult to visualize—premature metopic suture closure	++
Broad thumb	-	+++	+++	+++
Rocker-bottom feet	-	+++	+++	+++
Short femur	-	+	++	+++
Fetal growth—SGA-like	-	-	-	+
Low-lying conus medullaris	-	+	+	+
Increased pulmonary valve echogenicity with normal PSV	-	+	+	++
Bilateral cryptorchidism Shawl scrotum with excessive rugae	-	++	++	++

Table legend—ultrasound findings: “-” denotes absent; “+” visible; “++” clearly visible; “+++” markedly visible”; and “?” inconclusive/unclear finding.

**Table 2:** Prenatal ultrasound signs: cases in the literature. “x” means present/described in the study.

Findings	Report (Author and Year of Publications)														
	Greco et al. (2009) [14]	Bedeschi et al. (2014) [15]	Cardalliac et al. (2017) [18]	Van-Gils et al. (2019) [3]						D’Ambrosi et al. (2022) [4]	Wu et al. (2020) [19]	Zloto et al. (2024) [20]			
The gestational age (weeks) at detection of abnormal ultrasound findings	23	28	26	33	26	35	24	37	35	21	27	38	19	38	24
**Gene(s)**															
Prenatal															
*CREBBP*	x				x					ND			x		
*EP300*	x									ND					
Postnatal diagnosis (*CREBBP*)		x	x	x		x	x	x	x	ND	x	x		x	x
**Abnormal ultrasound**															
Central nervous system (CNS)															
Microcephaly		x		x				x	x						
Brachiacephalic head			x												
Cerebellar vermis hypoplasia/agenesis	x		x		x		x	x	x						
Corpus callosum dysgenesis		x											x	x	
Face and neck															
Moderate micrognathia	x					x			x					x	
Broad nasal bridge/short/absent nasal bone	x	x	x			x			x			x			
Low set ears													x		
Cleft lip and palate								x							
Thickened nuchal fold		x												x	
Extremities and skeletal system															
Broad, abducted thumbs and halluces/prominent broad big first toe/bifid thumb	x	x	x							x		x		x	x
Short long bones			x											x	
Cardiovascular system															
Abnormal course of the ductus venosus										x					
Hypoplasia of the pulmonary artery (HPA)				x											
Tetralogy of Fallot									x						
Ascending aorta and aortic arch—thin, narrow aorta and large oval hole											x				
Genitourinary system															
Unilateral/Bilateral renal hydronephrosis			x						x						
Duplication of the gallbladder				x									x		
Bilateral cryptorchidism Shawl scrotum with excessive rugae												x			
Gastrointestinal system															
Absent splenium													x		
Fetal annexes															
Polyhydramnios	x		x			x			x						
Fetal biometry															
Intrauterine growth restriction (IUGR)					x	x			x					x	

## Data Availability

The data used to support the findings of this study are available upon request to the corresponding author.

## Abbreviations

The following abbreviations are used in this manuscript:

ADHD	Attention deficit hyperactivity disorder
ASD	Autistic spectrum disorders
βHCG	Β human chorionic gonadotropin
HPA	Hypoplasia of the pulmonary artery
IUGR	Intrauterine growth restriction
MoM	Multiple of the median
MTHFR	Methylenetetrahydropholate reductase
PAPP-A	Pregnancy-associated plasma protein A
RSTS	Rubinstein–Taybi Syndrome
SNP array	Single-nucleotide polymorphism array
TF	Tetralogy of Fallot
